# Chemical and Sensory Profiling of Monovarietal Extra Virgin Olive Oils from the Italian Marche Region

**DOI:** 10.3390/antiox9040330

**Published:** 2020-04-17

**Authors:** Deborah Pacetti, Maria Chiara Boarelli, Rita Giovannetti, Stefano Ferraro, Paolo Conti, Barbara Alfei, Giovanni Caprioli, Massimo Ricciutelli, Gianni Sagratini, Donatella Fedeli, Rosita Gabbianelli, Dennis Fiorini

**Affiliations:** 1 Department of Agricultural, Food, and Environmental Sciences, Polytechnic University of Marche, Via Brecce Bianche, I-60131 Ancona, Italy; d.pacetti@staff.univpm.it; 2 School of Science and Technology, Chemistry Division, University of Camerino, V. S. Agostino 1, I-62032 Camerino (Macerata), Italy; mariachiara.boarelli@unicam.it (M.C.B.); rita.giovannetti@unicam.it (R.G.); stefano.ferraro@unicam.it (S.F.); paolo.conti@unicam.it (P.C.); 3 Agri-food Service Agency of Marche Region (ASSAM), Via dell’Industria 1, I-60027 Ancona, Italy; alfei_barbara@assam.marche.it; 4 School of Pharmacy, University of Camerino, V. S. Agostino 1, I-62032 Camerino (Macerata), Italy; giovanni.caprioli@unicam.it (G.C.); gianni.sagratini@unicam.it (G.S.); donatella.fedeli@unicam.it (D.F.); rosita.gabbianelli@unicam.it (R.G.); 5 HPLC-MS Laboratory, University of Camerino, V. S. Agostino 1, I-62032 Camerino (Macerata), Italy; massimo.ricciutelli@unicam.it

**Keywords:** Italian monovarietal extra virgin olive oil, chemical profile, sensory analysis, antioxidant activity, Ascolana tenera, Coroncina, Mignola, Piantone di Mogliano, Raggia

## Abstract

Chemical and sensory peculiarities of monovarietal extra virgin olive oils (MEVOOs) from the cultivars (cvs.) Ascolana tenera (ASC), Coroncina (COR), Mignola (MIG), Piantone di Mogliano (MOG), and Raggia (RAG) from Marche region (Italy) are investigated. Their polar phenolic substances and α-tocopherol are analysed through high performance liquid chromatography with different detectors. Volatile substances, fatty acid composition, and squalene are analysed by gas chromatography coupled to mass spectrometry (MS) and to the flame ionization detector, respectively. Total antioxidant activity and sensory analysis were also performed. MOG showed high squalene content (on average 0.88 ± 0.16 g/100 g), high relative amount of α-copaene among volatiles, and the highest oleic acid percentage. MIG had high α-tocopherol content (on average 350.0 ± 57.6 mg kg^−1^) and high α-farnesene in the volatile fraction. ASC showed the highest sensory quality and the lignan pinoresinol with higher concentration as compared to the other MEVOOs (*p* < 0.05), which resulted in a possible chemical marker for this cv. RAG was characterized by the sensory note of almond, which corresponds to its highest (*E*)-2-hexenal percentage. Sensory analysis and an antioxidant activity assay performed on a set of industrial extra virgin olive oils purchased in supermarkets, highlighted MEVOOs’ superiority from these points of view. Principal component analysis displays the main characteristics of the cvs. investigated.

## 1. Introduction

Extra virgin olive oil (EVOO) is considered the prince of edible oils due to its chemical composition rich of bioactive beneficial molecules and provides appreciated sensory characteristics [1]. In particular, the strength point of the chemical composition, is that both the major as well as the minor components have characteristics that are ideal in an oil. The saponifiable fraction is very well balanced, where oleic acid, which is a monounsaturated fatty acid, is largely the most represented fatty acid, which provides relatively high oxidative stability, even with a low content of saturated fatty acids. The unsaponifiable fraction contains a large number of different bioactive substances, which play a key role for the oil oxidative stability while promoting health effects and the sensory characteristics of the oil [2]. Several are the characteristics that recognize a food as being excellent. Italy is recognized as a leading country in terms of excellent food production, as demonstrated by the numerous cases of frauds often dealing with fake Italian foods [3]. The safeguard of the identity of food products having specific and special characteristics is allowed by the recognition of the chemical peculiarities characterizing high quality and niche products. Monovarietal EVOOs (MEVOOs) are in the foreground among Italian niche food products. The Italian germoplasm numbers over 800 cultivars (cvs.) [4] and the MEVOOs that can be obtained are products reflecting the characteristics of a country beyond the genetics. Furthermore, legislation helps to protect the origin of the products [5], which allows Protected Denominations of Origin (PDO) or Protected Geographical Indication (PGI) [4]. However, a deep knowledge of the chemical, and, thus, nutritional, peculiarities of MEVOOs is still lacking due to a recent growth of market for these excellent products [6]. Their detailed characterization leads to identify their key features and the relation with their quality. However, several cvs. that are spread in specific areas are not yet investigated extensively. 

Starting from this premise, this study aims to obtain a chemical and sensorial fingerprint of MEVOOs produced in the Marche region (Italy), from five cvs. (Raggia, Piantone di Mogliano, Ascolana tenera, Mignola, and Coroncina), selected among those more spread on that country and producing appreciated oils, often receiving prizes in regional, national, and sometimes international competitions in which their sensory properties are evaluated [7]. On oils obtained from the same varieties, other studies have been reported in literature. Rotondi et al. [4] reported average fatty acid, total phenol content, and sensory profiles of 16 MEVOOs oils out of the 177 varieties for which these parameters are also reported in the Italian monovarietal oils database [8]. Cecchi and Alfei [9] studied the volatiles profile of 11 different monovarietal oils from the Marche region (Italy) and correlated it with the sensory profile of the oils. Fiori et al. [10] evaluated the effect of malaxation time on the polyphenol content and composition of Ascolana tenera oil and its oxidation stability. The same variety is also investigated in a study [11] where it was introduced in Tunisia together with other varieties. The monovarietal oils produced were compared with that obtained from the autochthonous variety Chetoui by investigating several parameters related to oil quality. 

The present study, aiming to characterize the oils from the five cvs. Raggia (RAG), Piantone di Mogliano (MOG), Ascolana tenera (ASC), Mignola (MIG), and Coroncina (COR), applies to a relatively high number of different samples for each cultivar, obtained from different producers and from different areas crops, in each of the two years harvest (2015 and 2016). There were 6–12 oil samples for each year, for each cultivar. Thus, it may allow the attribution of some characteristics specifically to the olive cultivar. In particular, polar phenolic substances, volatile substances, fatty acid composition, tocopherol content, squalene content, antioxidant activity, and sensory properties, have been investigated. To the best of our knowledge, several of these parameters (e.g., squalene, α-tocopherol, antioxidant activity), have never been investigated in MEVOOs from these varieties.

Thus, the results of such a study contribute to fill the gap in the knowledge of features affecting nutritional, healthy, and sensory value of these oils and to better understand the relation between quality and chemical composition in EVOOs.

## 2. Materials and Methods

### 2.1. Reagents and Standards

The analytical standards of hydroxytyrosol, tyrosol, vanillic acid, oleuropein, luteolin, and apigenin were purchased from Extrasynthese (Genay, France). *p*-Coumaric acid, ferulic acid, pinoresinol, syringic acid, α-tocopherol, 2,2’-azinobis-(3-ethylbenzothiazoline-6-sulfonate) (ABTS), and Trolox were purchased from Sigma-Aldrich (Milan, Italy). High performance liquid chromatography (HPLC) -grade methanol, hexane, and isopropanol (IPA) were purchased from Sigma-Aldrich (Milan, Italy). Water (resistivity above 18 MΩ cm) was obtained from a Milli-Q SP Reagent Water System (Millipore, Bedford, MA, USA). Solvents and solutions were filtered through a 0.45-µm polytetrafluoroethylene (PTFE) filter from Supelco (Bellefonte, PA, USA). 

### 2.2. Sampling

A total of 79 MEVOOs from the cvs. ASC, COR, MIG, MOG, and RAG have been investigated. For each cultivar, several samples were provided by different producers from different areas of Marche region (Italy) for a total of 36 MEVOOs samples from the olive campaign 2015 (6 ASC, 7 COR, 7 MIG, 9 MOG, 7 RAG) and 43 (8 ASC, 7 COR, 8 MIG, 8 MOG, 12 RAG) from the olive campaign 2016. For each sample, two 0.75 L bottles were provided. One was used for the sensory analysis and another one was used for the chemical analysis. Additionally, a set of 24 industrial EVOOs purchased from local supermarkets were investigated for the sensory profile and for the antioxidant activity. According to their labels, 12 of these were produced in Italy with Italian olives and had a price between 8.29 and 25.8 euro/L and 12 were from EU countries, with a price between 3.78 and 7.49 euro/L. Samples were stored away from light and at 4 °C until the analysis. Before the analysis, samples were left reaching a temperature of 25 °C and gently homogenized. For each of the two years of sampling, oils were analysed in the period March-April after their arrival in the laboratory.

### 2.3. Fatty Acid Composition

Fatty acid methyl esters were obtained by reacting 5 mg of the oil dissolved in hexane (1 mL) with 2N potassium hydroxide in methanol (0.1 mL) in a 4-mL screw capped vial, which was shaken for 2 min with a vortex device. Then, 1.5 mL of 0.15 M acetic acid aqueous solution was added to quench the reaction and other 0.5 mL of hexane to allow a better separation of the two phases. The mixture was vortexed for 30 s and then centrifuged for 2 min at 5000 rpm. The upper hexane phase was then analysed in a gas chromatograph (GC) coupled with a flame ionization detector (FID). One µL was injected in split mode (ratio 1:30) in the hot split/splitless injector (T 260 °C) of a GC-FID (6850 Agilent Technologies, Santa Clara, CA, USA). The carrier gas was hydrogen produced by a generator (PGH2-250 from DBS Analytical Instruments, Vigonza, Italy). The initial gas flow in the column was 3.7 mL/min. Chromatographic column coating was 50% cyanopropylphenyl and 50% polydimethylsiloxane (DB-225-MS, length 30 m, 0.25 mm i.d., 0.25 μm film thickness, Agilent Technologies, Santa Clara, CA, USA). The oven temperature program was increased to 220 °C at a rate of 20 °C/min, maintained for 5 min, and then increased at 20 °C/min to 240 °C. The temperature was held for 1 min, which resulted in a total run time of 19 min. The FID temperature was 250 °C and air and hydrogen flows were 400 and 40 mL/min, respectively.

### 2.4. Quantification of Olive oil Polar Phenolic Compounds by HPLC-DAD-ESI/MS

A previously reported procedure [12] was followed to extract and analyse polar phenolic substances. Briefly, 0.5 g of oil were dissolved in 0.5 mL of hexane. The internal standard solution was added (20 µL of 100 mg L^−1^ syringic acid in methanol) and extracted with 4 × 0.5 mL of methanol: water (60:40, *v*/*v*). The hydro-alcoholic solutions were collected, washed with 1 mL of hexane to remove acylglycerols left, evaporated to dryness, and reconstituted with 0.25 mL of methanol before the analysis.

Analyses were performed using an HPLC Agilent 1100 (Santa Clara, CA, USA) with a diode–array detector (DAD) and a mass spectrometer detector (ion trap G2445D SL) equipped with an electrospray ionization (ESI) source in reported conditions [13]. A Synergi Polar reverse phase (250 × 4.6 mm, 4 m) analytical column from Phenomenex (Chesire, UK) was used with, as a mobile phase, water (A) and methanol/IPA 90:10 *v*/*v* with (B) each containing 0.1% formic acid, working in the gradient mode at a flow rate of 1 mL min−1, and injecting 10 µL of sample extract solution. Gradient used was: 0 min, 30% B, 0–40 min, 60% B, 40–50 min 95% B. In HPLC-DAD analysis, different wavelengths were monitored: 260 nm for vanillic acid, 280 nm for hydroxytyrosol, tyrosol, and secoiridoids derivatives, pinoresinol, acetoxypinoresinol, and syringic acid, 310 nm for *p*-coumaric acid, 325 nm for ferulic acid, 338 nm for apigenin, and 350 nm for luteolin. In HPLC-ESI–MS, the ion source was operated in a negative ionization (NI) mode and a mass analyser in full scan mode. Mass scan was performed in the range m/z 70–1100. The main secoiridoid derivatives were quantified using the calibration curve of tyrosol. Acetoxypinoresinol was quantified with the calibration curve of pinoresinol, while all the other phenolics quantified each one with its own calibration curve, as reported in [12].

### 2.5. Determination of α-Tocopherol

A 25 mg/mL solution of the oil sample in hexane was prepared and filtered through a 0.45-µm PTFE filter before the analysis by HPLC coupled to a fluorescence detector (HPLC-FLD), performed injecting 10 µL. The analytical column used was a Hypersil silica column (200 × 2.1 mm, 5 μm, from Thermo Fisher Scientific, Waltham, Massachusetts, USA). The mobile phase was hexane with 0.25% IPA, at a flow rate of 0.5 mL min^−1^. FLD was set with an excitation wavelength of 290 nm and an emission wavelength of 330 nm. For the quantification, a calibration curve (correlation coefficient R = 0.9992) was built by analyzing seven standard stock solutions of α-tocopherol in hexane at concentrations of 0.53–10.6 µg mL^−1^. 

### 2.6. Volatile Substances

A 1.5 g of oil in a screw cap vial with pierceable septum was conditioned at 40 °C for 10 min by stirring at 300 rpm with a small magnet. A solid-phase microextraction fibre coated with 50/30 µm divinylbenzene/carboxen/polydimethylsiloxane (DVB/CAR/PDMS), 1 cm long, was then exposed to the headspace of the sample for 30 min. Analytes were desorbed in the hot gas chromatograph injector kept at 260 °C by performing the injection in a splitless mode (4 min). The instrument used was a GC coupled to a mass spectrometer detector (Agilent 6850 GC-MSD 5973N, Agilent Technologies, Santa Clara, CA, USA). The separation was performed with a capillary column coated with polyethylene glycol (DB-WAX, length 60 m, internal diameter 0.25 mm, film thickness 0.25 µm, Agilent Technologies, Santa Clara, CA, USA). Carrier gas (helium) flow was 1.2 mL min^−1^, and the oven temperature program was: 40 °C held for 4 min, then ramped at 2.5 °C min^−1^ until 120 °C, and then ramped at 15 °C min^−1^ until 250 °C. Then it was held for 3.33 min. The transfer line was held at 250 °C, ion source (electron impact was at 70 eV) at 230 °C, and quadrupole at 150 °C. Mass scan range was 29–400 amu. Identification of eluted substances was done comparing the experimental retention indices, calculated referring to linear alkanes, with those reported in literature, and by comparing the experimental mass spectra with those of the NIST 08 library.

### 2.7. Squalene Analysis

Squalene was quantified in the samples by applying a reported procedure [14]. Furthermore, 15 mg of oil was dissolved in 1 mL of hexane, added 10 μL of internal standard solution (squalane in hexane at 10 mg mL^−1^), and subjected to transmethylation with methanolic potassium hydroxide solution (0.1 mL, 2 M). After stirring for 2 min, the reaction was quenched by adding saturated brine (1.5 mL). The hexane phase was directly analysed by GC coupled to a flame ionization detector (6850, Agilent Technologies, Santa Clara, CA, USA) by injecting 1 μL in the hot injector at 300 °C in a split mode (20:1 split ratio) using as chromatographic column of a 5% phenylpolydimethylsiloxane coated capillary column (HP-5, length 30 m, 0.32 mm i.d., 0.25 μm film thickness, Agilent Technologies, Santa Clara, CA, USA) with a run time of 5 min.

### 2.8. Total Antioxidant Activity (TAA) 

The total antioxidant activity of MEVOOs and industrial EVOOs was determined according to a reported procedure [15]. This test is based on the capacity of antioxidant compounds to quench the ABTS radical cation [2,2′- azinobis (3-ethylbenzothiazoline-6-sulfonic acid) diammonium salt], a blue/green chromophore absorbing at 734 nm. The decrease of absorbance is proportional to the antioxidants found in the sample. The ABTS radical cation solution was prepared by reacting 7 mM ABTS in water with 2.5 mM potassium sulphate. Ethanol was added to reach an absorbance of 0.7 ± 0.2 at 734 nm. The TAA was determined on oils 1:4 (*v*/*v*) diluted with hexane. Two mL of ABTS solution, mixed with 10 μL of olive oil solution was incubated at room temperature away from light for 10 min, and then read at 30 °C in a spectrophotometer at 734 nm. Solvent blanks were prepared for each assay. The absorbance decrease was referred to the calibration curve obtained in the presence of known concentrations of Trolox (6-hydroxy-2,5,7,8-tetramethylchroman-2-carboxylic acid), a vitamin E analogue, which is an antioxidant widely used as an antioxidant activity index. Results are reported in terms of Trolox concentrations (μM). 

### 2.9. Sensory Analysis

Sensory analysis was performed according to the procedure reported in the European Commission regulation n. 2568/1991 [16] and in its subsequent modifications. Analyses were performed by ASSAM-Marche, whose panel was authorized by the International Olive Council (IOC) until 2004, and then by the Ministry of Agricultural, Food, and Forestry Policies (MIPAAF). 

### 2.10. Statistical Analysis

Statistical analysis was performed to highlight significant differences between the oil groups investigated by means of one-way analysis of variance (ANOVA) and Tukey’s pairwise test using the software PAST [17]. Principal component analysis was performed by The Unscrambler X (Camo software, 2009–2012).

## 3. Results and Discussion

### 3.1. Fatty Acid Composition

**Figure 1:**
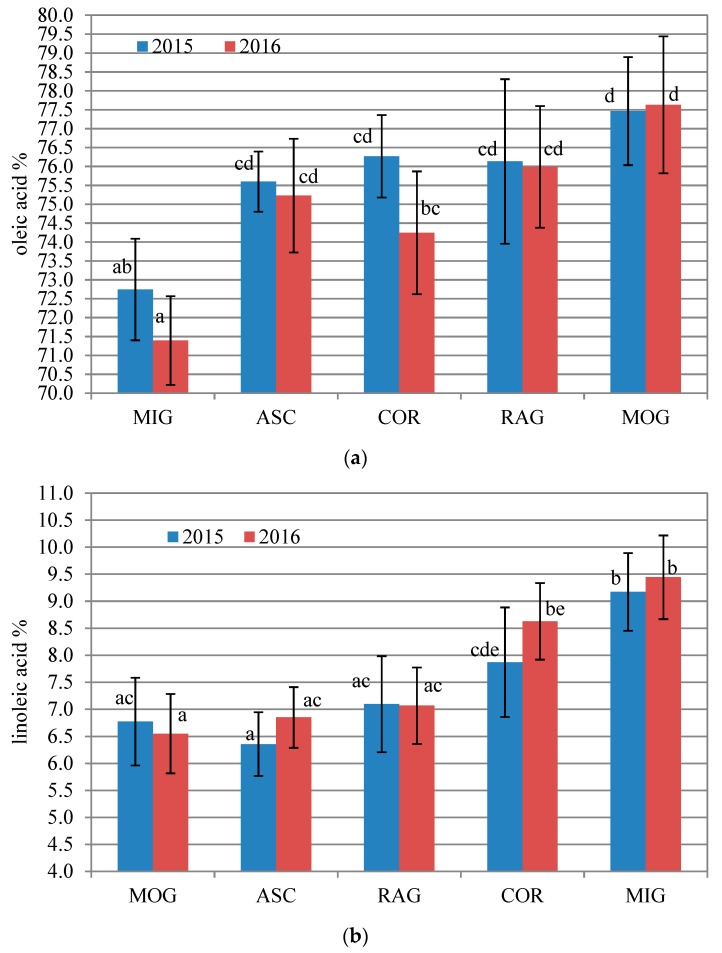
Oleic acid percentage (**a**) and linoleic acid percentage (**b**) in total fatty acid composition in Ascolana tenera (ASC), Coroncina (COR), Mignola (MIG), Piantone di Mogliano (MOG), and Raggia (RAG) monovarietal oils investigated, which was produced in 2015 and 2016. Bars indicate ± standard deviation. All different letters indicate significant differences (*p* < 0.05, One-way ANOVA, Tukey’s test for pairwise comparison) among the 10 oil sample groups investigated.

One of the major key strengths of olive oil composition is its very high percent content of oleic acid and low percent content of linoleic acid bound in the acylglycerol backbone, which makes olive oil highly resistant toward oxidation, by maintaining a low level of saturated fatty acid percentage content. In Figure 1, the average percentage content of oleic acid and linoleic acid in each cultivar investigated during the two-year study are reported. Some clear features of the cvs. investigated can be highlighted. In both the years investigated, 2015 and 2016, MIG is the weakest cultivar from this point of view. In fact, it has the lowest content of oleic acid (72.7 ± 1.3% in 2015 and 71.4 ± 1.2% in 2016) and the highest content of linoleic acid (9.2 ± 0.7% in 2015 and 9.4 ± 0.8% in 2016), differing significantly (*p* < 0.05) in almost all the cases from the other cvs. Differently, MOG has the best characteristics from this point of view, having both the years with the highest average value of the oleic acid percentage, which is almost identical in the two years (77.5 ± 1.4% and 77.6 ± 1.8%, in 2015 and 2016, respectively), and the lowest percentage content of linoleic acid (6.8 ± 0.8% and 6.5 ± 0.7%, in 2015 and 2016, respectively). These results are in total agreement with the study of Rotondi et al. [4] who reported results referring to a very high number of samples investigated. A general agreement was also found with results reported by Cecchi et al. [18] with the only exception of ASC for which they found a slightly lower percentage content of linoleic acid (4.2 ± 0.2% versus 6.6 ± 0.6% of the present study) and higher oleic acid percentage (79.8 ± 4.0% versus 75.6 ± 1.2% of the present study). The trend found for the different cvs. is very similar in the two years, and, considering that all the oils have been produced with olives generally harvested at the ideal ripening degree [19], the obtained results support the genetic reason for the trend found. The finding is in agreement with other studies [20,21], which allows us to recognize clear differences between the cvs. investigated. The effect of seasonality on the fatty acid composition was weak as shown by the absence of significant differences in the comparison between the two years in each of the cvs. investigated.

### 3.2. Polar Phenolic Substances

Polar phenolic compounds are key components for assessing the quality of an olive oil due to their contribution to sensory, healthy, and stability properties. Within these substances, great attention has been devoted in the last years to a sub-group of phenolics indicated as “hydroxytyrosol and its derivatives like oleuropein complex and tyrosol” [22], whose concentration, when exceeding 250 mg kg^−1^ of oil, allows the acknowledgement of the health claim on olive oil polar phenolic compounds. To the best of our knowledge, the composition of polar phenolic compounds in the monovarietal oils from cvs. investigated in the present study, have never been reported earlier with the only exception of ASC, whose oils, obtained from crops cultivated in Tunisia, have been studied from this point of view [11]. Among the 79 oils investigated in the present study, 70 resulted in a composition that allows the acknowledgement of the mentioned health claim. Thus, this shows high quality from this point of view, similarly to what was found by Antonini et al. [23]. These authors reported that all the Italian EVOOs investigated (28 from protected designation of origin consortia and 256 from little family farms all over the Italian country) had levels allowing the acknowledgement of the health claim. The cultivar with the highest total polar phenolic compounds content (largely represented, from 89.2% to 95.0%, by the substances considered by the health claim) resulted in RAG having an average total concentration of 434.2 ± 151.2 mg kg^−1^. The one with the lowest content was MOG with an average total concentration of 318.3 ± 123.3 mg kg^−1^ (Table 1). In addition, the average content of the total phenolic substances considered by the above-mentioned health claim does not vary significantly between the cvs. investigated. In 2015, it varies from 255.7 ± 112.5 mg kg^−1^ found in MOG to 443.4 ± 169.7 mg kg^−1^ found in RAG while, in 2016, from 346.0 ± 104.8 mg kg^−1^ in ASC to 395.7 ± 130.5 mg kg^−1^ in COR. However, in both years, RAG is one of the two cvs. with the highest content and MOG is one of the two with the lowest content. Considering the whole of the MEVOOs investigated, their average content of the phenolic substances considered by the health claim, is higher as compared to levels reported for EVOO blends commonly found in the large retail distribution market (supermarket) [24,25]. Interesting information to fingerprint the different varieties were provided mostly by minor phenolics, like phenolic acids, lignans, and flavonoids, which seem to be more characteristic for the cvs. investigated as compared to the major phenolic substances (secoiridoid derivatives). Statistical analysis indicated that the content of acetoxypinoresinol was significantly higher in RAG (*p* = 4.4 10^−9^ and 1.1 10^−7^ in 2015 and 2016 samples, respectively) than in each of the other cvs. investigated in both years. The finding is in agreement with other studies pointing at lignans as possible varietal markers [26]. Regarding phenolic acids, MOG and MIG had the highest content of *p*-coumaric acid and vanillic acid, respectively, in both years.

### 3.3. α-Tocopherol

The average content of α-tocopherol in the different cvs. investigated resulted in an identical trend in the two years. The cultivar having the highest content was MIG, with an average content of 295.9 ± 25.7 mg kg^−1^ in 2015 and of 397.3 ± 39.9 mg kg^−1^ in 2016, which was followed by ASC and then by COR, RAG, and MOG, which contained the lowest average amount: 192.3 ± 42.2 mg kg^−1^ in 2015 and 259.0 ± 44.2 mg kg^−1^ in 2016 (Table 1). Thus, the results indicate a clear relation of the tocopherol content with the cultivar, which was also shown in other studies [27,28,29]. The average content was higher in 2016 as compared to 2015 for each of the cvs. investigated with statistical significance at *p* < 0.05 for COR and MIG. Thus, this reveals an effect of the pedo-climatic, agronomic conditions, rather independent from the cultivar.

### 3.4. Volatile Substances

In this study, a selection of volatile molecules among those detected in the EVOOs investigated, has been considered, based on their recognized relation with positive or negative sensory attributes of an olive oil. Their relative quantification has been performed in terms of peak area percentage, and significant differences between the cvs. were highlighted in each of the two years investigated (Table 2), with some features seeming to be peculiar of the cultivar, as suggested by an identical trend in both years. Volatile substances are key compounds concerning the sensory properties of an EVOO where they can provide positive sensory notes as the green, fruity, almond and many other desirable attributes. However, some of the volatiles (e.g., ethanol, acetic acid, some aldehydes) are also responsible for negative sensory attributes of the oil (e.g., rancid, winey, yeast). Thus, the profile of the volatile substances can be related to the quality of an EVOO highlighting some peculiarities of the specific olive cultivar from which the oil has been obtained. The lipoxygenase pathway is the key biochemical process leading to formation of a series of volatile molecules, mainly having five or six carbons, that are aldehydes, alcohols, or their ester derivatives with acetic acid. (*E*)-2-Hexenal, which is one of the main compounds formed in the lipoxygenase pathway, is a key molecule in EVOO flavor, which contributes with sensory notes of green leaves, grass, and fruit and is commonly found to be the most abundant volatile detected. Other aldehydes are present in EVOO and their presence, referring in particular to longer chain aldehydes, as e.g., 2-heptenal, nonanal, and decanal [30], which is due to autoxidation phenomena leading the oil to lose its sensory and nutritional quality, beyond its safety. Pentene dimers are a group of isomers formed during lipoxygenase pathway, which have been associated with positive sensory attributes of the oil [31]. Terpenes (e.g., α-farnesene, α-copaene) are also commonly found in EVOO and their presence has been related to the olive cultivar, which has molecules already present in the olive fruit and are partially transferred to the oil during the production. α-Copaene is a tricyclic sesquiterpene whose presence in the fruit has been reported to contribute to an increase in the susceptibility of olive fruits to attack by *Bactrocera oleae* females [32]. Methanol and ethanol are produced to a large extent in undesirable fermentation processes occurring during prolonged olives storage before processing and higher levels are generally found in oils having lower quality [33,34,35]. In the present study, oils from the cultivar ASC were characterised by a high average percent of the C5 compounds 1-penten-3-one and 1-penten-3-ol, by (*Z*)-3-hexen-1-ol and 3-hexen-1-ol acetate and by a low relative percentage of (*E*)-2-hexenal (Table 2). MIG had a high percent of 3-hexen-1-ol and hexanol acetates, pentene dimers, and α-farnesene, which was highest in MIG in each of the two years even if statistical significance was not reached in 2016. On the other side, MIG was relatively poor of 1-penten-3-one and 1-penten-3-ol, (*E*)-2-hexenal, hexanal, and α-copaene. In both years investigated, RAG was the cultivar with the highest average percentage of (*E*)-2-hexenal, 82.2 ± 4.6% in 2015 and 72.1 ± 10.3% in 2016, which is significantly higher than its average percentage of content in MIG and in ASC in both years. The absolute content of the molecule, measured in terms of peak area units, has the same trend, with RAG having the highest average value, as compared to the other cvs. in both years, and differing significantly from MIG in both years. MOG variety was characterised by the highest percent content of α-copaene in each of the two years, with significant differences among the groups investigated, which points at α-copaene as a possible marker for the variety. The result is in agreement with the study of Cecchi and Alfei [9], where α-copaene percentage in the volatile composition of 11 different monovarietal oils investigated resulted in the highest value in MOG. The high content of this volatile could also possibly cause this cultivar to be more susceptible to the attack by *B. oleae* [32].

### 3.5. Squalene

**Figure 2:**
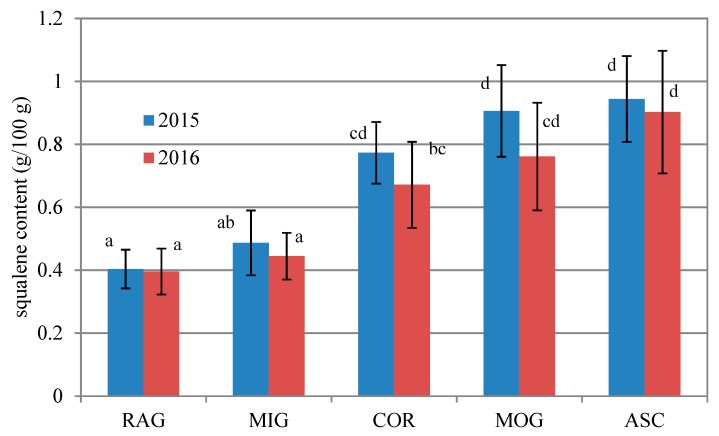
Average squalene content (g/100 g) in Ascolana tenera (ASC), Coroncina (COR), Mignola (MIG), Piantone di Mogliano (MOG), and Raggia (RAG) monovarietal oils investigated were produced in 2015 and 2016. Bars indicate ± standard deviation. All different letters indicate significant differences (*p* < 0.05, One-way ANOVA, Tukey’s test for pairwise comparison) among the 10 oil sample groups investigated.

Squalene is a triterpene having several beneficial properties and is found in a relatively high amount of olive oil [36]. Its content has been reported to depend markedly on the olive cultivar [37]. Thus, the present study, aiming to highlight the peculiarities of monovarietal oils, took into consideration the content of this bioactive substance, which was never investigated before in these cvs. The results (Figure 2) confirm a strong influence of variety on the squalene content found in the oils. The lowest average concentration (0.40 g/100 g) was found in RAG while the highest average value (0.92 g/100 g) was found in ASC. Together with MOG cultivar, it resulted in “very high content” varieties (>0.75 g/100 g) when considering the classification reported by Beltran et al. [37]. The same trend was found for each cultivar in the two years and, between the two years, no significant differences have been found, which further supports a genetic reason behind the squalene concentration trend found in the MEVOOs investigated [37].

### 3.6. Antioxidant Activity

**Figure 3:**
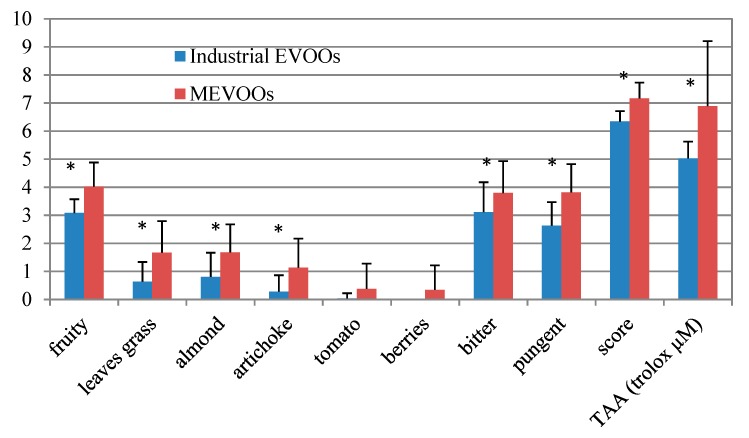
Comparison between the results obtained from sensory analysis and total antioxidant activity assay (TAA) of the 79 monovarietal extra virgin olive oils (MEVOOs) samples investigated (14 Ascolana tenera, 14 Coroncina, 15 Mignola, 17 Piantone di Mogliano, 19 Raggia) and 24 industrial extra virgin olive oils (EVOOs) purchased from supermarkets. Positive bars indicate standard deviation. Significant differences between Industrial EVOOs and MEVOOs are highlighted by the asterisk (*p* < 0.05, One-way ANOVA and Tukey’s test for a pairwise comparison).

The total antioxidant activity was determined in the 79 MEVOOs investigated in the present study when compared to that found in industrial EVOOs purchased in local supermarkets (Table 3, Figure 3). Results do not show significant differences among the five cvs. investigated (Table 3). The result is in agreement with that obtained from the HPLC analysis of the polar phenolic substances and with the content of secoiridoid derivatives, which are the most abundant polar phenolic compounds of EVOOs and mainly responsible for their antioxidant properties. Furthermore, it should be considered that the antioxidant activity is given by many different substances (e.g., polar phenolic substances, tocopherols, squalene, and others) and, within the varieties investigated, some were found to be rich of one (or more) of these antioxidants and poor in others while the opposite was true for other cvs. (e.g., MOG is very rich in squalene while it contains low concentration of polar phenolic substances. RAG contains a very high amount of polar phenolic substances and a low amount of squalene, and so forth). Moreover, the different response given by the reactant to the different antioxidant substances further decreases the capability of this test of differentiating oils from different cvs.. However, the antioxidant activity shown by the whole of the MEVOOs investigated, on average 6.9 ± 2.3 trolox µM, resulted in a significantly higher value (*p* < 0.05) as compared to that given by industrial EVOOs purchased from a large retail distribution market (on average, 5.0 ± 0.6 trolox µM), which demonstrates the generally higher quality of niche MEVOOs in terms of antioxidant activity as compared to industrial EVOOs (Figure 3).

### 3.7. Sensory Analysis

Sensory analysis was performed by an officially recognized panel, according to the procedure reported in the European Commission regulation n. 2568/91 [16] and subsequent modifications. Attributes evaluated were fruity, bitter, and pungent. However, others like e.g., the attributes of leaves/grass, artichoke/cardoon, almond, tomato, berries as well as the total score represent an indication of the overall sensory quality of the oil [25]. The oils investigated showed a generally higher level of quality in 2015 as compared to 2016 whereas, for each of the cvs. investigated, the positive attributes resulted in almost all of the cases being higher in 2015 as compared to 2016 in several cases with significant differences (*p* < 0.05, Table 3). This can be explained by the environmental conditions of the two years. In 2016, higher humidity and lower temperature favoured the attack of the olives by *Bactrocera oleae*, which resulted in a generally lower intensity of the positive sensory attributes and, thus, a generally lower quality of the oils produced as compared to 2015 oils. This finding is also in agreement with the general trend observed in Italy in these two years and reported in the national database [7]. However, independently from seasonal variations, some peculiarities of the cvs. investigated are clearly shown by the results obtained. ASC in both the years results in a cultivar with the highest average value being received for the fruity attribute, and was confirmed to be characterized by a typical note of tomato [9], highlighted in a negligible amount in the cvs.. COR presents high values for all of the attributes with the only exceptions of tomato and berries attributes resulting in peculiarity of ASC and MIG, respectively. The high score for pungency, bitterness, and artichoke/cardoon in both years is in agreement with its high relative polar phenolic substances content, which is well known to provide the oil with these sensory notes. RAG has a peculiar sensory profile. In both the years, it received the highest average score for the attributes of the almond, which can be plausibly explained with its very high content of (*E*)-2-hexenal, known to be a volatile molecule providing this sensory note. However, RAG was the variety that showed the highest variability in two years. It was the only one where all the parameters investigated (with the only exception of berries and tomato that are peculiar of specific cvs.) have scores significantly lower in 2016 as compared to 2015. The peculiar attribute of berries, specifically related to MIG, correlated with the content of hexyl acetate, found to be particularly high in MIG as compared to the other cvs. in both years. Hexyl acetate is a volatile substance recognized to provide fruity notes and previously associated with the berry note in wines [38]. A set of industrial EVOOs purchased from local supermarkets was also analysed in order to assess differences with MEVOOs investigated in the present study, which are artisanal products and generally recognized as having higher quality. A pronounced difference is found for each of the parameters considered in the sensory analysis (Figure 3). All of them resulted in significantly higher values in MEVOOs as compared to industrial EVOOs with the only exception of “tomato” and “berries” due to the fact that these attributes are specific of ASC and MIG, respectively. Thus, the results are negligible in the 79 MEVOOs investigated.

### 3.8. Principal Component Analysis

**Figure 4:**
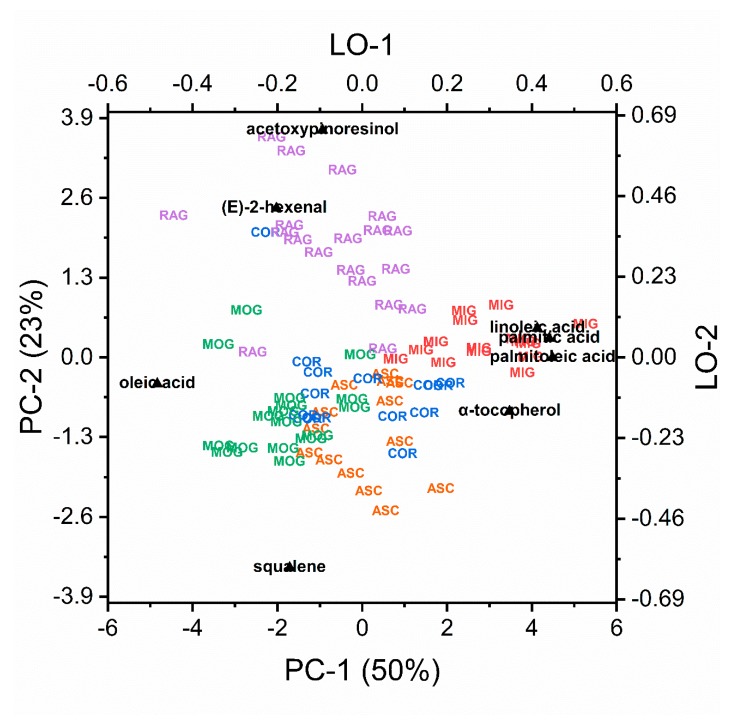
Biplot with projection of PC-1 vs PC-2, from PCA analysis on a subset of chemical parameters selected in order to optimize the olive cultivar separation in the monovarietal oils investigated (Ascolana tenera: ASC, Coroncina: COR, Mignola: MIG, Piantone di Mogliano: MOG, Raggia: RAG). PC: principal component, PCA: principal component analysis, LO: loading.

The previously discussed features have been studied by Principal Component Analysis (PCA) to highlight the relations between them, using only auto-scaled variables. Several variables show very low correlation with the most important PCs. Then, only the most important were retained: palmitic, palmitoleic, oleic, linoleic acids, α-tocopherol, (*E*)-2-hexenal, acetoxypinoresinol, and squalene. PCA on the selected variables permits a raw grouping of the samples, based on the different olive cvs., as shown in Figure 4.

This figure shows that (*E*)-2-hexenal, acetoxypinoresinol, and squalene divide the groups along PC-2 axis, that is, RAG and MIG from the others. Along PC-1, there is a mild separation between MIG and the others. In this direction, there are high loadings for palmitoleic, palmitic, and linoleic acids, low loading of oleic acid, and high loading of α-tocopherol. PCA shows that few parameters, among the many investigated, are correlated with the olive cultivar. We can conclude that the analysis of the chemical parameters of the monovarietal oils analyzed permits to differentiate them, but are likely due to the relatively low number of samples the predictive ability is poor. The phenolic fraction of the analyzed parameters seems to be the most related to the varieties while the other parameters are related to quality (seasonal dependence) alone or confused with varietal differences.

## 4. Conclusions

The presented study highlights peculiar chemical features of MEVOOs produced in Marche region (Italy) and are much appreciated for their sensory properties. The study shows that, behind the sensory properties, peculiar chemical features characterize these MEVOOs, which are mostly highlighted for the first time in this study, and which point out why MEVOOs can be considered as functional foods. In most of the cases investigated here can be acknowledged with the European Food Safety Authority health claim on olive oil polar phenolic compounds, which support the healthy properties of these EVOOs. Beyond the interesting compositional features highlighted in each of the varieties studied, the MEVOOs investigated, as a whole, showed a significant difference in terms of sensory quality with respect to industrial oils. This process demonstrated their superiority both from the point of view of their antioxidant activity as well as for their sensory quality.

## Figures and Tables

**Table 1:** Average content of polar phenolic substances and of α-tocopherol ± standard deviation (SD) in the investigated monovarietal oils (ASC: Ascolana tenera, COR: Coroncina, MIG: Mignola, MOG: Piantone di Mogliano, RAG: Raggia) produced in 2015 and 2016 and in the total 2015/2016 samples.

	Hydroxytyrosol	Tyrosol	Vanillic Acid	*p*-Coumaric Acid	Ferulic Acid	Secoiridoid Derivatives	Pinoresinol	Acetoxy pinoresinol	Luteolin	Apigenin	Total Polar Phenolics	α-Tocopherol
	Average (mg kg^−1^) ± SD											
ASC 2015 (*n* = 6)	5.46 ^ab^ ± 2.14	6.00 ± 2.97	0.33 ± 0.16	0.10 ^ab^ ± 0.03	0.03 ^a^ ± 0.02	353.23 ± 108.89	12.49 ^a^ ± 5.55	5.98 ^a^ ± 7.54	2.30 ^ab^ ± 0.59	0.90 ^b^ ± 0.34	387.80 ^ab^ ± 182.13	270.91 ^adc^ ± 39.50
COR 2015 (*n* = 7)	4.06 ^a^ ± 1.62	4.67 ± 1.39	0.32 ± 0.17	0.11 ^a^ ± 0.04	0.03 ^a^ ± 0.03	340.19 ± 109.02	4.72 ^b^ ± 1.56	8.86 ^a^ ± 8.34	3.24 ^b^ ± 1.16	1.25 ^b^ ± 0.31	367.45 ^ab^ ± 113.52	^#^ 229.92 ^bc^ ± 16.48
MIG 2015 (*n* = 7)	8.75 ^b^ ± 5.16	9.24 ± 6.16	0.49 ± 0.23	0.09 ^ab^ ± 0.03	0.03 ^a^ ± 0.02	282.14 ± 113.72	^#^ 6.48 ^b^ ± 1.58	8.13 ^a^ ± 1.69	2.10 ^ab^ ± 0.47	0.49 ^a^ ± 0.15	317.94 ^ab^ ± 121.26	^#^ 295.94 ^a^ ± 25.69
MOG 2015 (*n* = 9)	2.38 ^a^ ± 1.53	5.11 ± 1.22	0.46 ± 0.21	0.12 ^a^ ± 0.04	0.08 ^b^ ± 0.02	248.22 ± 112.33	7.63 ^ab^ ± 2.33	5.31 ^a^ ± 4.32	1.49 ^a^ ± 0.95	0.94 ^b^ ± 0.30	271.73 ^a^ ± 112.89	192.35 ^b^ ± 42.15
RAG 2015 (*n* = 7)	^#^ 6.79 ^ab^ ± 2.79	6.86 ± 2.59	0.34 ± 0.14	^#^ 0.05 ^b^ ± 0.02	0.05 ^ab^ ± 0.02	429.75 ± 172.33	^#^ 3.83 ^b^ ± 0.54	35.47 ^b^ ± 10.34	3.08 ^b^ ± 1.47	1.25 ^b^ ± 0.18	487.48 ^b^ ± 175.89	215.98 ^bc^ ± 47.51
ASC 2016 (*n* = 8)	5.01 ± 2.86	6.24 ± 3.76	0.31 ± 0.19	0.12 ± 0.04	0.06 ± 0.02	334.77 ± 104.17	18.45 ^a^ ± 14.09	8.94 ^a^ ± 9.22	3.04 ^ab^ ± 0.99	0.92 ^ab^ ± 0.24	377.90 ± 103.23	356.68 ^bc^ ± 81.67
COR 2016 (*n* = 7)	5.32 ± 2.16	5.78 ± 2.78	0.35 ± 0.25	0.14 ± 0.06	0.05 ± 0.03	384.59 ± 127.68	6.27 ^b^ ± 1.25	5.52 ^a^ ± 4.65	3.61 ^ab^ ± 0.76	1.20 ^b^ ± 0.19	412.86 ± 133.15	^§^ 351.16 ^bc^ ± 50.76
MIG 2016 (*n* = 8)	6.37 ± 1.98	5.53 ± 2.41	0.52 ± 0.47	0.09 ± 0.05	0.05 ± 0.02	339.73 ± 105.57	^§^ 9.70 ^ab^ ± 1.74	10.30 ^a^ ± 5.03	2.51 ^ab^ ± 0.63	0.59 ^a^ ± 0.28	375.47 ± 105.31	^§^ 397.26 ^b^ ± 36.94
MOG 2016 (*n* = 8)	4.94 ± 5.46	7.47 ± 6.65	0.35 ± 0.16	0.18 ± 0.12	0.07 ± 0.04	335.27 ± 112.60	10.29 ^ab^ ± 5.77	9.03 ^a^ ± 6.41	2.06 ^a^ ± 0.75	0.92 ^ab^ ± 0.29	370.68 ± 119.33	259.01 ^a^ ± 44.19
RAG 2016 (*n* = 12)	^§^ 3.83 ± 2.08	6.20 ± 3.66	0.44 ± 0.26	^§^ 0.13 ± 0.09	0.07 ± 0.04	349.47 ± 131.68	^§^ 5.98 ^b^ ± 2.31	31.77 ^b^ ± 12.38	4.05 ^b^ ± 2.14	1.10 ^b^ ± 0.41	403.13 ± 132.91	286.66 ^ac^ ± 67.26
ASC 2015/2016 (*n* = 14)	5.21 ^ab^ ± 2.50	5.40 ± 2.00	0.32 ± 0.17	0.11 ± 0.04	0.05 ^a^ ± 0.02	342.68 ± 102.44	15.90 ^a^ ± 11.32	7.67 ^a^ ± 8.36	2.72 ^ab^ ± 0.90	0.91 ^b^ ± 0.27	376.97 ± 103.64	322.37 ^bc^ ± 79.12
COR 2015/2016 (*n* = 14)	4.69 ^ab^ ± 1.95	5.13 ± 1.98	0.34 ± 0.21	0.12 ± 0.05	0.04 ^a^ ± 0.03	362.39 ± 116.37	5.49 ^b^ ± 1.58	7.19 ^a^ ± 6.71	3.42 ^bc^ ± 0.96	1.23 ^c^ ± 0.25	390.16 ± 121.19	294.58 ^bc^ ± 72.97
MIG 2015/2016 (*n* = 15)	7.48 ^a^ ± 3.86	6.53 ± 3.17	0.51 ± 0.36	0.09 ± 0.04	0.04 ^a^ ± 0.02	312.85 ± 109.54	8.20 ^b^ ± 2.31	9.29 ^a^ ± 3.89	2.32 ^ab^ ± 0.58	0.54 ^a^ ± 0.22	348.63 ± 112.83	349.98 ^b^ ± 60.85
MOG 2015/2016 (*n* = 17)	3.58 ^b^ ± 3.99	6.13 ± 3.36	0.40 ± 0.19	0.15 ± 0.09	0.07 ^b^ ± 0.03	289.19 ± 117.74	8.88 ^b^ ± 4.37	7.06 ^a^ ± 5.57	1.76 ^a^ ± 0.88	0.93 ^b^ ± 0.29	318.30 ± 123.26	219.01 ^a^ ± 53.43
RAG 2015/2016 (*n* = 19)	4.92 ^ab^ ± 2.72	8.29 ± 6.26	0.40 ± 0.22	0.10 ± 0.08	0.06 ^ab^ ± 0.03	379.05 ± 148.59	5.19 ^b^ ± 2.12	33.14 ^b^ ± 11.52	3.70 ^c^ ± 1.94	1.16 ^bc^ ± 0.34	434.21 ± 151.18	260.62 ^ac^ ± 68.88

Statistical analysis compares the five different cultivars in the same year (2015 and 2016) and in the two years 2015/2016 (significant differences at *p* < 0.05 are highlighted by different letters). The same cultivar is also compared in the two different years and significant differences at *p* < 0.05 are highlighted by different symbols.

**Table 2:** Average percent composition in terms of peak area percentage ± standard deviation (SD) of selected volatile compounds analysed by head-space solid-phase microextraction and gas chromatography coupled to mass spectrometry, in the investigated monovarietal oils (ASC: Ascolana tenera, COR: Coroncina, MIG: Mignola, MOG: Piantone di Mogliano, RAG: Raggia) produced in 2015 and 2016 and in the total 2015/2016 samples.

	Methanol	Ethanol	3-Pentanone	1-Penten-3-one	4,8-Dimethyl-1,7-Nonadiene	1-Penten-3-ol	(*E*)-2-Hexenal	Hexyl Acetate	Hexanal	Octanal	(*Z*)-3-Hexen-1-ol Acetate	(*Z*)-2-Penten-1-ol	6-Methyl-5-Hepten-2-one	1-Hexanol	(Z)-3-Hexen-1-ol	(*E*)-2-Hexen-1-ol	Nonanal	α-Copaene	1-Octanol	α-Farnesene	Total Pentene Dimers
	Area % ± SD																				
ASC 2015 (*n* = 6)	^#^3.54^ab^ ± 2.96	^#^6.11 ± 6.46	^#^1.68^ab^ ± 1.25	3.83 ± 1.96	1.07 ± 0.70	1.91 ^b^ ± 1.61	49.84 ^a^ ± 17.41	0.67 ^ac^ ± 0.15	4.45 ^abc^ ± 3.78	0.68 ± 0.76	6.23 ± 1.63	^#^0.18 ± 0.18	nd	2.53 ^ab^ ± 1.83	^#^11.15 ^b^ ± 9.84	0.81 ± 0.53	2.92 ± 2.66	0.68 ^ab^ ± 0.75	0.31 ± 0.51	0.21 ^a^ ± 0.37	4.94 ^ab^ ± 4.49
COR 201 (*n* = 7)	^#^3.23^a^ ± 1.59	3.07 ± 2.72	0.75^a^ ± 0.58	3.16 ± 1.87	1.10 ± 0.64	0.90 ^ab^ ± 0.42	64.96 ^ab^ ± 11.84	0.51 ^bc^ ± 0.49	2.38 ^c^ ± 1.50	0.28 ± 0.35	^#^1.89 ± 1.25	^#^0.32 ± 0.53	nd	1.63 ^a^ ± 1.94	3.04 ^a^ ± 2.08	1.83 ± 1.37	2.25 ± 3.18	0.25 ^b^ ± 0.24	0.08 ± 0.14	0.35 ^a^ ± 0.20	8.02 ^a^ ± 3.46
MIG 2015 (*n* = 7)	^#^1.82^ab^ ± 1.06	6.74 ± 5.02	^#^2.43^b^ ± 1.41	1.41 ± 1.11	1.23 ± 0.33	0.83 ^ab^ ± 0.34	47.60 ^a^ ± 22.76	1.12 ^a^ ± 0.44	1.97 ^c^ ± 1.20	0.32 ± 0.11	4.71 ± 2.47	^#^0.10 ± 0.09	0.13 ± 0.15	5.36 ^b^ ± 4.27	^#^4.72 ^ab^ ± 2.96	9.11 ± 1.74	1.28 ± 0.53	0.04 ^b^ ± 0.10	nd	0.98 ^b^ ± 0.58	8.09 ^a^ ± 2.22
MOG 2015 (*n* = 9)	1.75^ab^ ± 1.16	3.04 ± 3.69	^#^1.20^ab^ ± 0.82	2.42 ± 1.17	^#^0.60 ± 0.33	1.19 ^ab^ ± 0.68	64.68 ^ab^ ± 11.02	0.03 ^b^ ± 0.09	6.59 ^b^ ± 3.60	0.20 ± 0.19	0.27 ± 0.49	^#^0.14 ± 0.13	nd	2.12 ^ab^ ± 1.15	^#^7.25 ^a^ ± 3.75	^#^2.28 ± 1.72	0.97 ± 0.84	1.00 ^a^ ± 0.56	nd	0.08 ^a^ ± 0.12	3.80 ^ab^ ± 1.66
RAG 2015 (*n* = 7)	0.60 ^b^ ± 0.38	1.87 ± 1.45	0.52^a^ ± 0.52	1.63 ± 1.03	^#^0.50 ± 0.18	0.57 ^a^ ± 0.30	^#^82.91 ^b^ ± 4.60	0.09 ^b^ ± 0.13	1.84 ^c^ ± 0.50	0.20 ± 0.10	0.43 ± 0.34	^#^0.10 ± 0.06	nd	1.41 ^a^ ± 0.97	^#^0.84 ^a^ ± 0.18	2.27 ± 9.97	1.43 ± 1.28	0.16 ^b^ ± 0.14	0.06 ± 0.08	0.08 ^a^ ± 0.14	^#^2.47 ^b^ ± 1.63
ASC 2016 (*n* = 8)	^§^1.01 ± 1.46	^§^0.43 ± 0.21	^§^0.44 ± 0.64	3.16 ± 3.58	1.29 ± 1.66	0.94 ± 0.79	53.26 ^a^ ± 13.23	1.08 ^abc^ ± 0.83	4.03 ± 1.21	0.69 ± 0.83	4.94 ± 3.43	^§^0.78 ± 0.45	0.10 ± 0.15	1.66 ± 0.86	^§^4.61 ^c^ ± 1.04	3.92 ± 3.88	8.51 ± 8.43	0.14 ^a^ ± 0.33	0.69 ± 0.99	0.15 ± 0.17	8.14 ± 3.90
COR 2016 (*n* = 7)	^§^1.43 ± 0.64	2.36 ± 2.76	0.57 ± 0.54	2.51 ± 1.31	1.42 ± 1.45	0.77 ± 0.39	58.58 ^ab^ ± 2.79	1.18 ^ac^ ± 0.86	3.21 ± 1.86	0.12 ± 0.28	^§^6.22 ± 3.51	^§^1.23 ± 0.50	nd	1.58 ± 0.81	3.32 ^bc^ ± 1.13	2.55 ± 1.62	2.72 ± 2.98	0.02 ^a^ ± 0.05	0.09 ± 0.21	0.17 ± 0.19	9.52 ± 3.70
MIG 2016 (*n* = 8)	^§^0.62 ± 0.44	2.22 ± 2.50	^§^0.38 ± 0.42	1.03 ± 0.80	1.25 ± 1.00	0.39 ± 0.37	52.36 ^a^ ± 15.19	1.54 ^a^ ± 0.75	3.63 ± 2.15	0.54 ± 1.00	6.48 ± 4.05	^§^0.73 ± 0.45	0.13 ± 0.17	1.68 ± 0.93	^§^2.63 ^ac^ ± 0.84	2.31 ± 1.67	8.32 ± 9.52	nd	0.28 ± 0.42	0.95 ± 0.73	12.75 ± 7.60
MOG 2016 (*n* = 8)	1.07 ± 0.61	1.48 ± 1.85	^§^0.29 ± 0.37	1.67 ± 1.28	^§^1.11 ± 1.28	0.48 ± 0.39	66.85 ^ab^ ± 7.14	0.03 ^d^ ± 0.09	3.84 ± 2.47	nd	0.33 ± 0.74	^§^0.64 ± 0.48	0.24 ± 0.40	2.08 ± 1.95	^§^3.05 ^cd^ ± 1.62	^§^4.51 ± 4.60	2.57 ± 3.30	0.65 ^b^ ± 0.32	nd	0.85 ± 1.28	8.24 ± 5.88
RAG 2016 (*n* = 12)	0.66 ± 0.52	1.00 ± 0.84	0.42 ± 0.54	1.43 ± 0.71	^§^2.17 ± 1.11	0.48 ± 0.14	^§^72.11 ^b^ ± 10.35	0.34 ^bcd^ ± 0.25	2.61 ± 1.23	0.30 ± 0.68	0.69 ± 0.77	^§^0.70 ± 0.35	0.03 ± 0.07	1.59 ± 1.52	^§^1.15 ^a^ ± 0.55	3.54 ± 2.82	3.92 ± 8.92	0.01 ^a^ ± 0.05	0.21 ± 0.49	0.18 ± 0.20	^§^6.46 ± 2.05
ASC 2015/2016 (*n* = 14)	2.10 ^ab^ ± 2.49	2.32 ± 4.38	0.91 ± 1.07	3.38 ^b^ ± 3.05	1.21 ± 1.33	1.32 ^b^ ± 1.21	51.88 ^b^ ± 14.01	0.95 ^bc^ ± 0.69	4.1 ^abc^ ± 2.21	0.69 ^b^ ± 0.77	5.37 ^a^ ± 2.93	0.55 ± 0.47	0.06 ± 0.13	1.99 ± 1.32	6.79 ^c^ ± 6.12	2.72 ± 3.37	6.36 ± 7.20	0.35 ^a^ ± 0.57	0.55 ± 0.84	0.17 ^ab^ ± 0.25	6.91 ^ab^ ± 4.27
COR 2015/2016 (*n* = 14)	2.33 ^a^ ± 1.49	2.74 ± 2.64	0.67 ± 0.55	2.86 ^ab^ ± 1.60	1.25 ± 1.05	0.84 ^ab^ ± 0.40	62.08 ^bc^ ± 9.17	0.82 ^cd^ ± 0.74	2.76 ^c^ ± 1.66	0.20 ^ab^ ± 0.32	3.89 ^abc^ ± 3.31	0.74 ± 0.69	nd	1.61 ± 1.47	3.17 ^ab^ ± 1.64	2.16 ± 1.47	2.67 ± 3.00	0.15 ^a^ ± 0.21	0.08 ± 0.17	0.27 ^ab^ ± 0.21	8.71 ^ab^ ± 3.50
MIG 2015/2016 (*n* = 15)	1.18 ^ab^ ± 0.98	3.96 ± 4.16	1.17 ± 1.36	1.17 ^a^ ± 0.91	1.24 ± 0.79	0.56 ^a^ ± 0.41	50.43 ^b^ ± 17.68	1.38 ^b^ ± 0.66	2.99 ^c^ ± 1.97	0.46 ^ab^ ± 0.78	5.80 ^a^ ± 3.52	0.48 ± 0.47	0.13 ± 0.15	3.21 ± 3.27	3.43 ^abc^ ± 2.11	4.93 ± 6.83	5.62 ± 8.10	0.02 ^a^ ± 0.06	0.17 ± 0.35	0.96 ^b^ ± 0.65	10.95 ^b^ ± 6.39
MOG 2015/2016 (*n* = 17)	1.43 ^ab^ ± 0.98	2.36 ± 3.05	0.78 ± 0.78	2.07 ^ab^ ± 1.24	0.84 ± 0.91	0.86 ^ab^ ± 0.66	65.69 ^ac^ ± 9.15	0.03 ^a^ ± 0.09	5.31 ^b^ ± 3.33	0.10 ^a^ ± 0.17	0.30 ^d^ ± 0.60	0.37 ± 0.42	0.11 ± 0.29	2.10 ± 1.51	5.29 ^bc^ ± 3.58	3.32 ± 3.44	1.72 ± 2.39	0.84 ^b^ ± 0.48	nd	0.44 ^a^ ± 0.93	5.87 ^a^ ± 4.63
RAG 2015/2016 (*n* = 19)	0.64 ^b^ ± 0.46	1.32 ± 1.15	0.46 ± 0.52	1.51 ^a^ ± 0.82	1.56 ± 1.20	0.51 ^a^ ± 0.21	76.09 ^a^ ± 9.98	0.25 ^ac^ ± 0.24	2.33 ^c^ ± 1.07	0.26 ^ab^ ± 0.54	0.60 ^bcd^ ± 0.65	0.48 ± 0.40	0.02 ± 0.06	1.52 ± 1.32	1.03 ^a^ ± 0.47	3.07 ± 2.50	3.00 ± 7.12	0.07 ^a^ ± 0.11	0.15 ± 0.39	0.14 ^a^ ± 0.18	4.99 ^a^ ± 2.71

Statistical analysis compares the five different cultivars in the same year (significant differences at *p* < 0.05 are highlighted by different letters) and the same cultivar in two different years (significant differences at *p* < 0.05 are highlighted by different symbols). nd: not detected (peak area values below 500000 units).

**Table 3:** Results from sensory analysis (average score of each sensory attribute and average overall score) and from antioxidant activity assay (total antioxidant activity (TAA) in terms of trolox (µM) ± standard deviation (SD) in the investigated monovarietal oils (ASC: Ascolana tenera, COR: Coroncina, MIG: Mignola, MOG: Piantone di Mogliano, RAG: Raggia) produced in 2015 and in 2016, and in the total 2015/2016 samples.

	Sensory Analysis									Antioxidant Activity
	Fruity	Bitter	Pungent	Leaves/Grass	Almond	Artichoke	Tomato	Berries	Score	TAA (Average Trolox µm ± SD)
	Average ± SD									
ASC 2015 (*n* = 6)	^#^ 5.34 ^a^ ± 0.30	4.76 ± 0.41	^#^ 4.67 ^ab^ ± 0.30	^#^ 2.95 ^a^ ± 0.72	1.71 ± 1.02	^#^ 2.29 ± 0.97	^#^ 2.60 ± 0.47	nd	7.76 ^a^ ± 0.27	8.58 ± 1.81
COR 2015 (*n* = 7)	^#^ 4.44 ^b^ ± 0.45	4.55 ± 0.70	^#^ 4.65 ^ab^ ± 0.48	2.37 ^bd^ ± 0.53	2.28 ± 0.62	1.84 ± 0.90	0.27 ± 0.72	0.23 ± 0.61	7.26 ^ab^ ± 0.38	8.10 ± 1.74
MIG 2015 (*n* = 7)	^#^ 4.21 ^b^ ± 0.51	4.16 ± 0.52	3.78 ^a^ ± 0.62	1.18 ^b^ ± 0.86	^#^ 1.36 ± 0.79	0.57 ± 0.73	nd	1.95 ± 1.23	7.12 ^ab^ ± 0.66	7.81 ± 1.49
MOG 2015 (*n* = 9)	4.20 ^b^ ± 0.58	3.73 ± 0.87	4.05 ^ab^ ± 0.93	2.17 ^bd^ ± 0.93	1.82 ± 0.92	1.31 ± 0.99	0.19 ± 0.57	0.23 ± 0.70	7.06 ^b^ ± 0.50	7.50 ± 2.04
RAG 2015 (*n* = 7)	^#^ 4.79 ^ab^ ± 0.49	^#^ 4.93 ± 0.79	^#^ 4.83 ^b^ ± 0.48	^#^ 2.51 ^acd^ ± 0.79	^#^ 3.04 ± 0.44	^#^ 1.90 ± 1.05	0.21 ± 0.54	nd	^#^ 7.55 ^ab^ ± 0.27	9.59 ± 2.86
ASC 2016 (*n* = 8)	^§^ 4.06 ± 1.16	3.36 ± 1.54	^§^ 3.37 ± 1.15	^§^1.47 ± 1.50	1.21 ± 0.98	^§^0.76 ± 0.87	^§^1.03 ± 1.45	nd	7.07 ± 0.85	5.84 ± 1.37
COR 2016 (*n* = 7)	^§^ 3.59 ± 0.55	3.76 ± 0.94	^§^ 3.74 ± 0.22	1.69 ± 1.21	1.59 ± 0.95	1.29 ± 1.21	0.16 ± 0.43	nd	7.13 ± 0.41	6.20 ± 2.33
MIG 2016 (*n* = 8)	^§^ 3.39 ± 0.42	3.66 ± 0.88	3.36 ± 0.66	0.53 ± 0.69	^§^0.56 ± 0.50	0.24 ± 0.46	nd	1.21 ± 1.39	7.18 ± 0.52	5.47 ± 2.09
MOG 2016 (*n* = 8)	3.66 ± 0.83	3.36 ± 1.12	3.33 ± 1.12	1.25 ± 0.83	1.65 ± 0.76	1.07 ± 1.00	nd	nd	6.96 ± 0.54	6.03 ± 1.72
RAG 2016 (*n* = 12)	^§^ 3.30 ± 0.69	^§^ 2.74 ± 1.10	^§^ 3.13 ± 1.24	^§^ 0.94 ± 0.69	^§^ 1.73 ± 1.05	^§^ 0.71 ± 0.70	nd	nd	^§^ 6.89 ± 0.59	5.40 ± 1.94
ASC 2015/2016 (*n* = 14)	4.70 ± 0.73	4.06 ± 0.97	4.02 ± 0.72	2.10 ^a^ ± 1.41	1.46 ± 1.00	1.53 ± 0.92	1.81 ± 0.96	nd	7.41 ± 0.56	7.02 ± 2.06
COR 2015/2016 (*n* = 14)	4.01 ± 0.50	4.15 ± 0.82	4.19 ± 0.35	2.03 ^a^ ± 0.97	1.94 ± 0.78	1.57 ± 1.06	0.22 ± 0.58	0.12 ± 0.31	7.19 ± 0.39	7.15 ± 2.21
MIG 2015/2016 (*n* = 15)	3.80 ± 0.46	3.91 ± 0.70	3.57 ± 0.64	0.84 ^b^ ± 0.82	0.96 ± 0.65	0.40 ± 0.59	nd	0.98 ± 0.62	7.15 ± 0.59	6.56 ± 2.14
MOG 2015/2016 (*n* = 17)	3.93 ± 0.71	3.54 ± 1.00	3.69 ± 1.02	1.74 ^ab^ ± 0.98	1.74 ± 0.84	1.19 ± 1.00	0.09 ± 0.28	0.12 ± 0.35	7.01 ± 0.52	6.81 ± 1.99
RAG 2015/2016 (*n* = 19)	4.05 ± 0.59	3.84 ± 0.94	3.98 ± 0.86	1.68 ^ab^ ± 1.08	2.39 ± 0.74	1.31 ± 0.88	0.10 ± 0.27	nd	7.22 ± 0.43	6.94 ± 3.06

For each sensory parameter, statistical analysis compares the five different cultivars with each other in the same year (2015 and 2016) and in the two years 2015/2016. Significant differences at *p* < 0.05 are highlighted by different letters. The same cultivar is also compared in the two different years (significant differences at *p* < 0.05 are highlighted by different symbols). and: not detected.
